# EEG sensorimotor rhythms’ variation and functional connectivity measures during motor imagery: linear relations and classification approaches

**DOI:** 10.7717/peerj.3983

**Published:** 2017-11-08

**Authors:** Carlos A. Stefano Filho, Romis Attux, Gabriela Castellano

**Affiliations:** 1 Neurophysics group, “Gleb Wataghing” Institute of Physics, University of Campinas, Campinas, São Paulo, Brazil; 2 Brazilian Institute of Neuroscience and Neurotechnology, Brazil; 3 Department of Computer Engineering and Industrial Automation, School of Electrical and Computer Engineering, University of Campinas, Campinas, São Paulo, Brazil

**Keywords:** Brain-computer interface, Electroencephalography, BCI, EEG, Motor imagery, MI, Graph theory, Functional brain networks

## Abstract

Hands motor imagery (MI) has been reported to alter synchronization patterns amongst neurons, yielding variations in the mu and beta bands’ power spectral density (PSD) of the electroencephalography (EEG) signal. These alterations have been used in the field of brain-computer interfaces (BCI), in an attempt to assign distinct MI tasks to commands of such a system. Recent studies have highlighted that information may be missing if knowledge about brain functional connectivity is not considered. In this work, we modeled the brain as a graph in which each EEG electrode represents a node. Our goal was to understand if there exists any linear correlation between variations in the synchronization patterns—that is, variations in the PSD of mu and beta bands—induced by MI and alterations in the corresponding functional networks. Moreover, we (1) explored the feasibility of using functional connectivity parameters as features for a classifier in the context of an MI-BCI; (2) investigated three different types of feature selection (FS) techniques; and (3) compared our approach to a more traditional method using the signal PSD as classifier inputs. Ten healthy subjects participated in this study. We observed significant correlations (*p* < 0.05) with values ranging from 0.4 to 0.9 between PSD variations and functional network alterations for some electrodes, prominently in the beta band. The PSD method performed better for data classification, with mean accuracies of (90 ± 8)% and (87 ± 7)% for the mu and beta band, respectively, versus (83 ± 8)% and (83 ± 7)% for the same bands for the graph method. Moreover, the number of features for the graph method was considerably larger. However, results for both methods were relatively close, and even overlapped when the uncertainties of the accuracy rates were considered. Further investigation regarding a careful exploration of other graph metrics may provide better alternatives.

## Introduction

Motor imagery (MI) has been investigated as a tool for aiding in specific situations, such as motor rehabilitation (Vries & Mulder, 2007; Mulder, 2007; Stevens & Stoykov, 2003), in particular, of hand movements after a stroke (Kaiser et al., 2011; Buch et al., 2012); improving athletic performance in sports (Mizuguchi et al., 2012); and complementing musical practice (Lotze, 2013; Brown & Palmer, 2013). In particular, hands-MI has been a well-exploited strategy in the brain-computer interface (BCI) community (Sivakami & Devi, 2015; Cheng et al., 2004; Neuper et al., 2005; Pfurtscheller & Neuper, 2001; Halder et al., 2011; Obermaier et al., 2001).

BCIs are systems that aim to control an external device using the brain as a direct communication channel. To do so, a BCI must be capable of recording the brain signals through some technique, processing them and classifying them according to the user’s intent. Electroencephalography (EEG) signals are the most used for BCI applications, although other techniques, such as functional magnetic resonance imaging (fMRI) (Berman et al., 2011; Hermes et al., 2011; Halder et al., 2013), near-infrared spectroscopy (NIRS) (Sitaram et al., 2007; Kanoh et al., 2009) and magnetoencephalography (MEG) (Mellinger et al., 2007; Lin et al., 2013) may be used.

MI has been reported to alter synchronization patterns amongst neurons, resulting in variations on the EEG signal regarding its power spectral density (PSD) in specific frequency bands (Pfurtscheller & Neuper, 1997). More specifically, MI induces event-related synchronizations (ERSs; and, thus, the PSD increases) in the γ frequency range (above 30 Hz) in the ipsilateral (to the MI) hemisphere and event-related desynchronizations (ERDs; and, thus, the PSD decreases) in the µ(7–13 Hz) and β (13–30 Hz) bands in the contralateral hemisphere (Al-ani & Trad, 2011; Pfurtscheller & Aranibar, 1977; Pfurtscheller, 1999; Neuper & Pfurtscheller, 1999). Therefore, a relatively well-established approach when studying MI-based EEG-BCIs has been to use the signals’ PSD as features for discriminating between MI tasks (Sivakami & Devi, 2015; Cincotti et al., 2001; Pfurtscheller & Neuper, 2001; Cheng et al., 2004).

Although features from spectral and temporal domains of the EEG signal have been successfully used in assessing MI data classification, recent studies have indicated that understanding the relationships between the recorded signals and their spatial locations in the form of functional brain connectivity may assist to improve the existing techniques (Hamedi, 2016). This understanding may also aid in overcoming limitations of the more traditional MI-BCIs, such as the inconsistency of MI responsive frequency bands across subjects (Kam, Suk & Lee, 2013) and the non-uniformity of ERDs and ERSs occurrences due to MI, which can vary their location depending on the subject (and even for the same subject, between distinct sections) (Asensio-Cubero, Gan & Palaniappan, 2013). These factors impose great challenges regarding the stability of features for classification of MI tasks and, thus, it becomes necessary to study other strategies that may be less sensitive to these variations.

In the present work, our goal was to understand if there exists any linear relationship (according to Pearson’s correlation) between PSD variations induced by MI and variations in functional connectivity measures. Moreover, we explored the feasibility of using elements from a functional connectivity matrix as features for a classifier, aiming to distinguish between left and right hands’ MI. In addition, we explored three different types of feature selection (FS) techniques, to analyze how these procedures can affect the classification outcome in the BCI scheme. Finally, we also compared our approach to a more traditional method by using the signal PSD as input for data classification.

## Materials and Methods

### Dataset and number of subjects

Data of left and right hands MI from a 64 channel EEG were obtained from the Physionet’s open database (Goldberger et al., 2000; Schalk et al., 2004), from which acquisitions from 10 subjects were analyzed in this study.

Data were acquired at a 160 Hz sampling rate. Experimental protocol consisted of randomly alternating blocks of task (right or left hand MI) or rest periods. Each block lasted approximately 4 s (for further details of the acquisition protocol, please refer to Schalk et al., 2004). Three runs were recorded for each subject, and each run contained between seven and eight blocks of each MI tasks, depending on the recording. To increase the quantity of available samples for our classifier, we analyzed them all as an ensemble, with each sample consisting of the whole 4 s block. For each subject, a total of 24 samples were available for training and testing the classifier.

### Data pre-processing

Data were filtered in the two frequency bands of interest most prominent in MI studies: µ(7–13 Hz) and β (13–30 Hz), using the standard FIR (finite impulse response) filter (Rabiner & Gold, 1975) of EEGLab (Delorme & Makeig, 2004), a MATLAB suite. This filtering operation would not have been necessary if we were to focus our analyses only on the signals PSD. However, since the signal time domain was considered to build the graphs, its component frequencies could influence calculations of the corresponding adjacency matrices. Thus, the filtering process enabled us to build specific graphs for each frequency band.

To attenuate common artifacts arising at all channels at the same time, data were spatially filtered using a CAR (common average removal) filter (Ludwig et al., 2009). Our reason for choosing this specific filter was three-folded: (1) CAR has been related to decreasing volume conduction effects, which is of particular interest for scalp EEG functional connectivity studies (Thakor & Tong, 2009; Yuksel & Olmez, 2016; Brunner et al., 2016). (2) Although more complex spatial filters have also been suggested (Thakor & Tong, 2009; Yuksel & Olmez, 2016; Brunner et al., 2016), a previous work has shown that the CAR filter provided similar MI classification results to more sophisticated techniques under specific feature selection situations (Uribe et al., 2016). Since in this work we were also interested in this issue, using the CAR filterin as a first approach was suggested by this finding. (3) Studies of the electrode referencing effect on the functional connectivity outcome have shown that using the average of the electrodes’ signal is one of the least distortive approacheswhen compared to other strategies, such as using the Cz electrode or the mastoids (Chella et al., 2016) (note that applying the CAR filter re-references the electrodes’ signals to their average).

### Graphs construction

**Figure 1:**
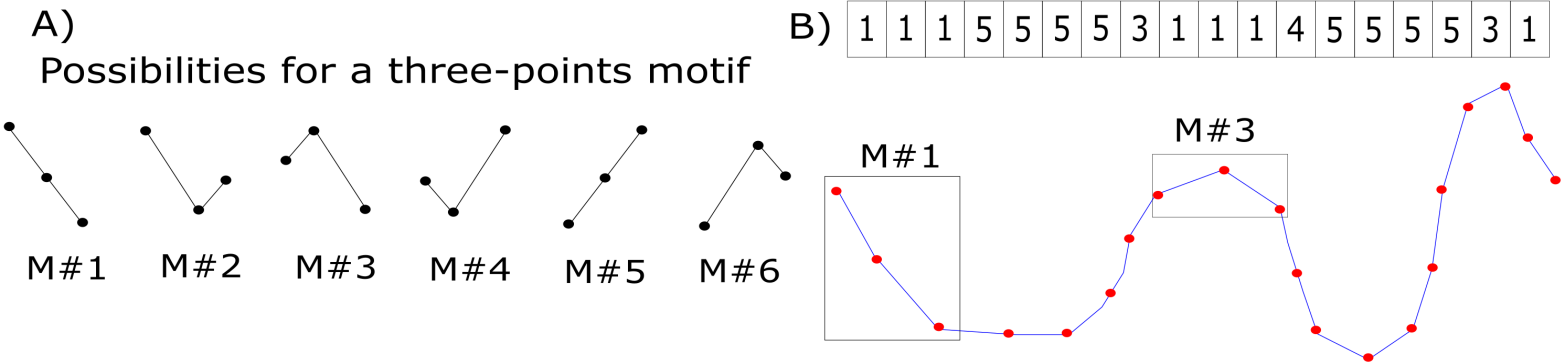
Motifs’ synchronization labeling. (A) Labels corresponding to possible variations of three data points. (B) Example of an original EEG time series (blue curve with the red points representing sampled data points) translated into a motifs series (labels). Two segments within the rectangular boxes are displayed as examples of motifs labels #1 and #3. On the top of the figure, the complete motif series for this sample signal is showed.

Graphs connectivity matrices were calculated using the motifs’ synchronization method (Rosario et al., 2015). The general principle is to divide the original time-series into smaller ensembles of data points (here we used three data points, as suggested by (Rosario et al., 2015)) and to label these new patterns according to Fig. 1A. This translates the original recorded EEG series into a new, labeled one—the motifs series (Fig. 1B). See, for example, the sample signal of Fig. 1B (blue curve with red dots corresponding to the sampled data points). The time-series is analyzed within a three-points sliding window, and the segment in this window is labeled accordingly. This process is performed for the time series of all electrodes. Due to the highly noisy nature of the EEG signal, we did not consider the possibility of two adjacent signal samples having the same value (see Fig. 1A).

The next step is to define a similarity coefficient between electrodes *i* and *j* (*s*_*ij*_) (i.e., between their time series). We calculated this by counting the number of coincidences (*N*) amongst their motif series’ elements and normalizing the result by the series’ length (*L*); that is:

(1) \begin{eqnarray*}{s}_{ij}= \frac{N}{L} .\end{eqnarray*}

Under this scheme, *s*_*ij*_ is normalized within the range [0,1]. To avoid losing important information by arbitrarily thresholding our graph’s edges for binarizing them, we chose to work with weighted graphs.

In summary, *s*_*ij*_ represents the *i*, *j*-th element of the functional connectivity matrix (FCM) related to the electrophysiological brain activity measured by EEG.

The motifs’ synchronization method should partially compensate for the volume conduction problem, since only the direction of change of the signal is considered, not contemplating effects that directly influence the signal’s magnitude. Moreover, since the construction of the functional networks was performed after the CAR filter was applied, the volume conduction issue should already have been at least partially accounted for.

### Features extraction, selection and data classification

Features of two distinct methods were tested, obtained either from the signal PSD or the connectivity approach, represented by the FCM.

The PSDs were estimated via Welch’s transform (Welch, 1967), and values from multiple frequencies were gathered for each electrode for further use by the classifier algorithm. More specifically, all frequencies of each band’s range were contemplated, collecting PSD values at unity steps. Therefore, each electrode yielded 7 and 18 frequencies (features) in the mu and beta band, respectively. In the case of the FCM, each one of its elements ( *s*_*ij*_) could be selected as a feature.

To study the effect of FS methods on the outcome classification results, we analyzed three approaches: (1) we designed a wrapper that should find the optimal set of electrodes, which would maximize the classifier’s outcome response. The wrapper’s search ended when no improvement occurred after four consecutive iterations. The other two FS strategies consisted of using a Pearson’s (2) or a Fisher’s (3) filter (Duch, 2006), combined with the wrapper described in (1). The basic idea of these filters is to rank the best attributes to be selected for classification according to a specific criterion. Pearson’s filter estimates how strongly a feature and its labeled-class are correlated by using Pearson’s linear correlation (Campbell & Swinscow, 1996). On the other hand, Fisher’s filter ranks attributes based on the criterion of the Fisher’s discriminant (McLachlan, 2004); that is, on the ratio between the difference of means and variances across data classes for that specific feature.

A least squares-based linear discriminant analysis (LDA) was used for MI data classification. This method was chosen due to its simplicity and robustness. Moreover, it has been commonly employed in BCI research, with results as good as those of other more complex classifiers (see, e.g., Carvalho et al., 2015).

All classification tests were performed using the leave-one-out scheme.

### Correlations between PSD and FCM

To investigate if any linear relationship between ERDs due to the hands MI tasks and variations on the FCM (when compared to the rest condition) could be observed, we performed analyses using Pearson’s correlation (Campbell & Swinscow, 1996).

Relative variations (either as ERDs or ERSs) of the signal’s PSD at a given frequency *f* on electrode *i* at a specific task block *t* (Δ*PSD*(*f*)_*i*,*t*_) were estimated as:

(2) \begin{eqnarray*}\Delta PSD(f)_{i,t}= \frac{PSD(f)_{i,r}-PSD(f)_{i,t}}{PSD(f)_{i,r}} .\end{eqnarray*}

In Eq. (2), *PSD*(*f*)_*i*,*r*_ represents the PSD value for electrode *i* and frequency *f* during an average of rest blocks. Note that a positive value for Δ*PSD*(*f*)_*i*,*t*_ indicates an ERD occured, whereas a negative one expresses an ERS.

Since each one of the 64 graph nodes contained information about other 63 connections, analyzing the *s*_*ij*_ values directly would require an elevated computational cost. Therefore, our correlation analyses were estimated indirectly by studying how the weighted degree of each node varied according to the Δ*PSD* magnitude, rather than the *s*_*ij*_ themselves. In doing so, we hypothesized that alterations in the functional networks (represented by *s*_*ij*_) should yield variations in the weighted degree for the graph nodes.

For a weighted graph, such as the ones built here, the weighted degree for a node *i* (*W*_*i*_) can be calculated as (Zhang et al., 2012):

(3) \begin{eqnarray*}{W}_{i}=\sum _{j}{s}_{ij}.\end{eqnarray*}

Variations relative to rest periods were then estimated for the weighted degree similarly to Eq. (2):

(4) \begin{eqnarray*}\Delta {W}_{i,t}= \frac{{W}_{i,r}-{W}_{i,t}}{{W}_{i,r}} .\end{eqnarray*}

In Eq. (4), indices *t* and *r* refer to a task block and the average of rest blocks, respectively.

Therefore, our correlation analyses considered the values of Δ*PSD* and Δ*W* for all trials of each electrode.

## Results

**Figure 2:**
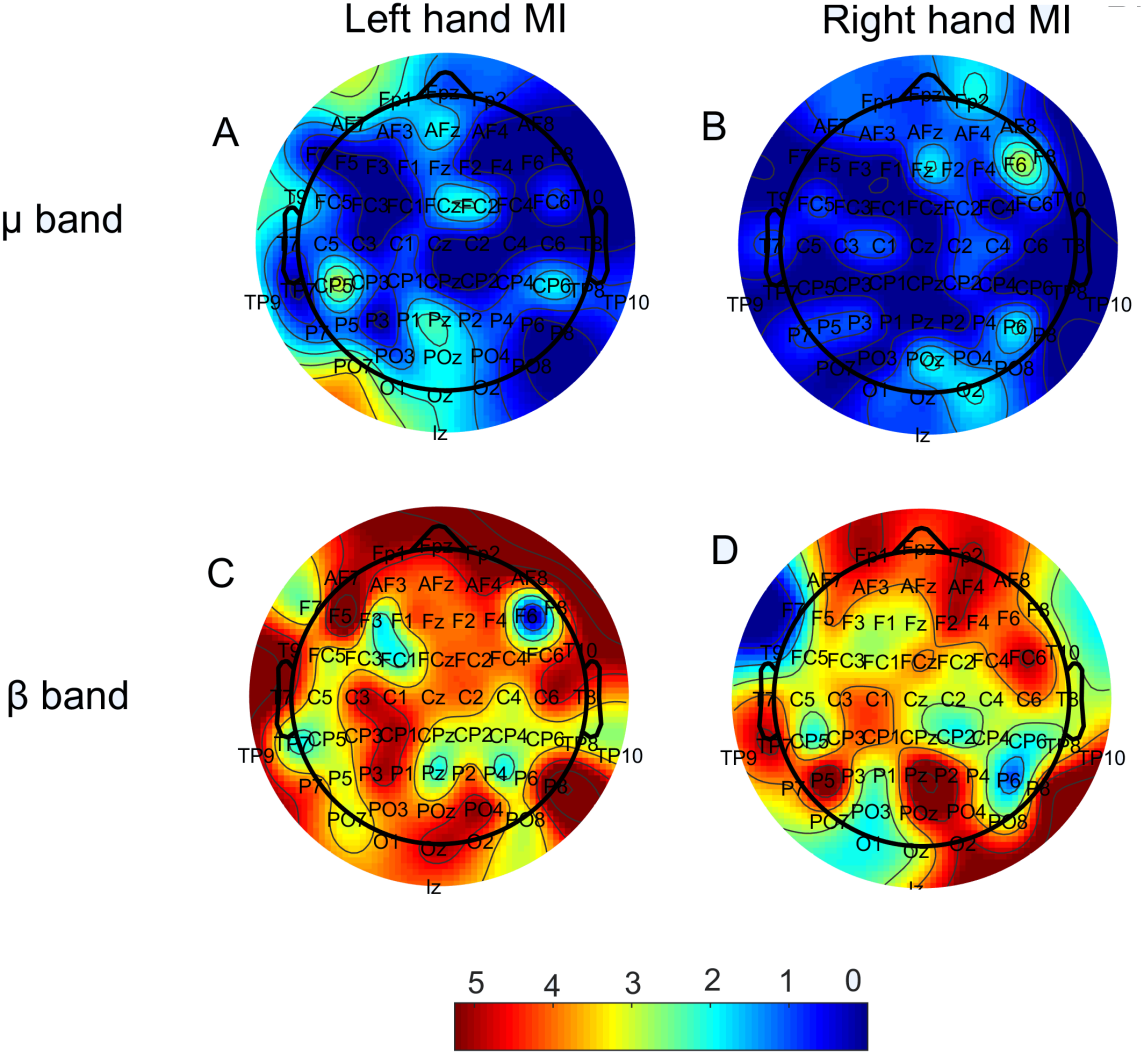
Number of times each electrode showed a significant correlation (*p* < 0.05) between the ERD relative to rest blocks (Δ*PSD*) and the degree variation on the respective node (Δ*W*). Both µ(upper row) and β (lower row) bands are present, as well as both MI tasks. (A) Left hand MI, mu band; (B) Right hand MI, mu band. (C) Left hand MI, beta band; (D) Right hand MI, beta band. Maximum numbers of occurrences on the same node were five (mu band) and seven (beta band).

Figure 2 displays the number of times each node presented a significant correlation (*p* < 0.05) between Δ*PSD* and Δ*W*. This result is shown as a colormap over the electrodes disposition, with colors closer to red indicating a larger value. Analyses were performed individually for each subject. Results in Fig. 2 show the sum of occurrences for all subjects; thus, they can be seen as frequency maps.

**Figure 3:**
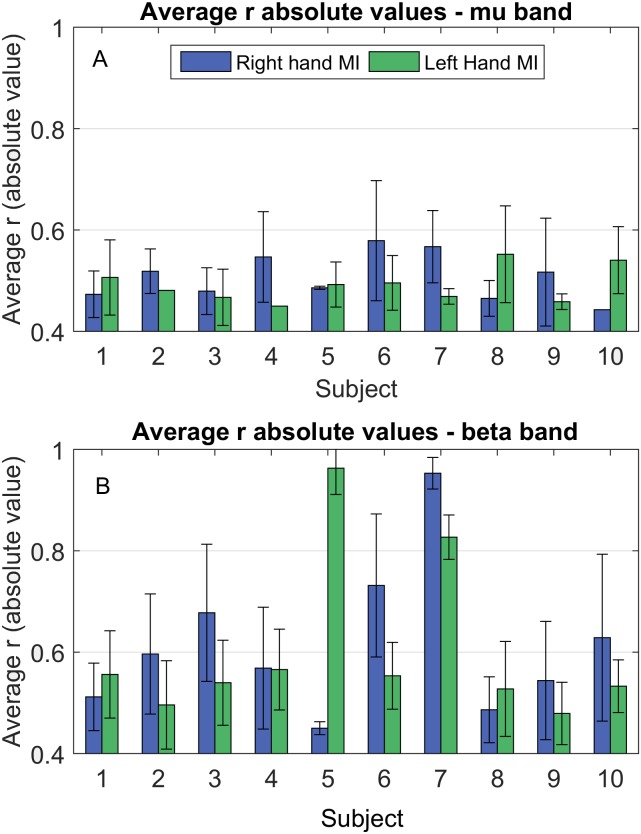
Average correlation coefficient (*r*) absolute values between Δ*PSD*(*f*)_*i*,*t*_ and Δ*W*_*i*,*t*_, during hands MI for mu (Fig. 3A) and beta (Fig. 3B) bands. Blue: right hand MI; green: left hand MI. Lack of error bars represents situations where only one correlation was observed.

Figure 3 displays bar plots of the absolute value for the Pearson correlation coefficient (*r*) for each subject, between Δ*PSD* and Δ*W*, during each MI task and for both frequency bands.

Table 1 displays classification accuracy rates for both bands. Each FS scheme is also shown, labeled according to the ‘Materials and Methods’ section: (1) wrapper only, (2) Pearson filter + wrapper, (3) Fisher filter + wrapper. All results are shown individually for each subject and averaged across all individuals with the respective standard deviation (std).

**Table 1:** Accuracy rates (%) for the different feature types tested. Results are shown individually and averaged across all subjects. (1) Wrapper only, (2) Pearson filter + wrapper, (3) Fisher filter + wrapper. Highest accuracy rates for a given subject and feature type are bold marked.

Accuracy rates (%)							
Frequency band	Subject	Feature					
		Δ*PSD*			*s*_*ij*_		
		(1)	(2)	(3)	(1)	(2)	(3)
µ(7–13 Hz)	S1	**100**	77	80	80	**82**	77
	S2	**100**	70	70	**93**	75	82
	S3	**89**	68	70	**86**	70	73
	S4	**93**	77	82	73	**84**	80
	S5	**83**	67	69	**93**	79	71
	S6	76	74	**79**	76	**81**	76
	S7	**98**	**98**	86	**91**	86	82
	S8	**91**	77	68	**80**	77	75
	S9	**82**	73	75	73	**80**	75
	S10	**86**	81	83	**86**	81	83
	**Mean ± std**	**90 ± 8**	**76 ± 9**	**76 ± 7**	**83 ± 8**	**80 ± 5**	**77 ± 4**

	Subject	Feature					
		Δ*PSD*			Δ*W*		
		(1)	(2)	(3)	(1)	(2)	(3)
β (13–30 Hz)	S1	**82**	80	**82**	**82**	80	**82**
	S2	**98**	77	82	**89**	84	84
	S3	**82**	75	75	**93**	84	82
	S4	**91**	77	80	**86**	80	80
	S5	**86**	71	60	**79**	74	76
	S6	**79**	69	71	**81**	76	69
	S7	**95**	68	61	**91**	75	77
	S8	**91**	77	68	80	**89**	73
	S9	**82**	80	70	**75**	73	73
	S10	**83**	69	81	71	79	**83**
	**Mean ± std**	**87 ± 7**	**74 ± 5**	**73 ± 8**	**83 ± 7**	**79 ± 5**	**78 ± 5**

**Figure 4:**
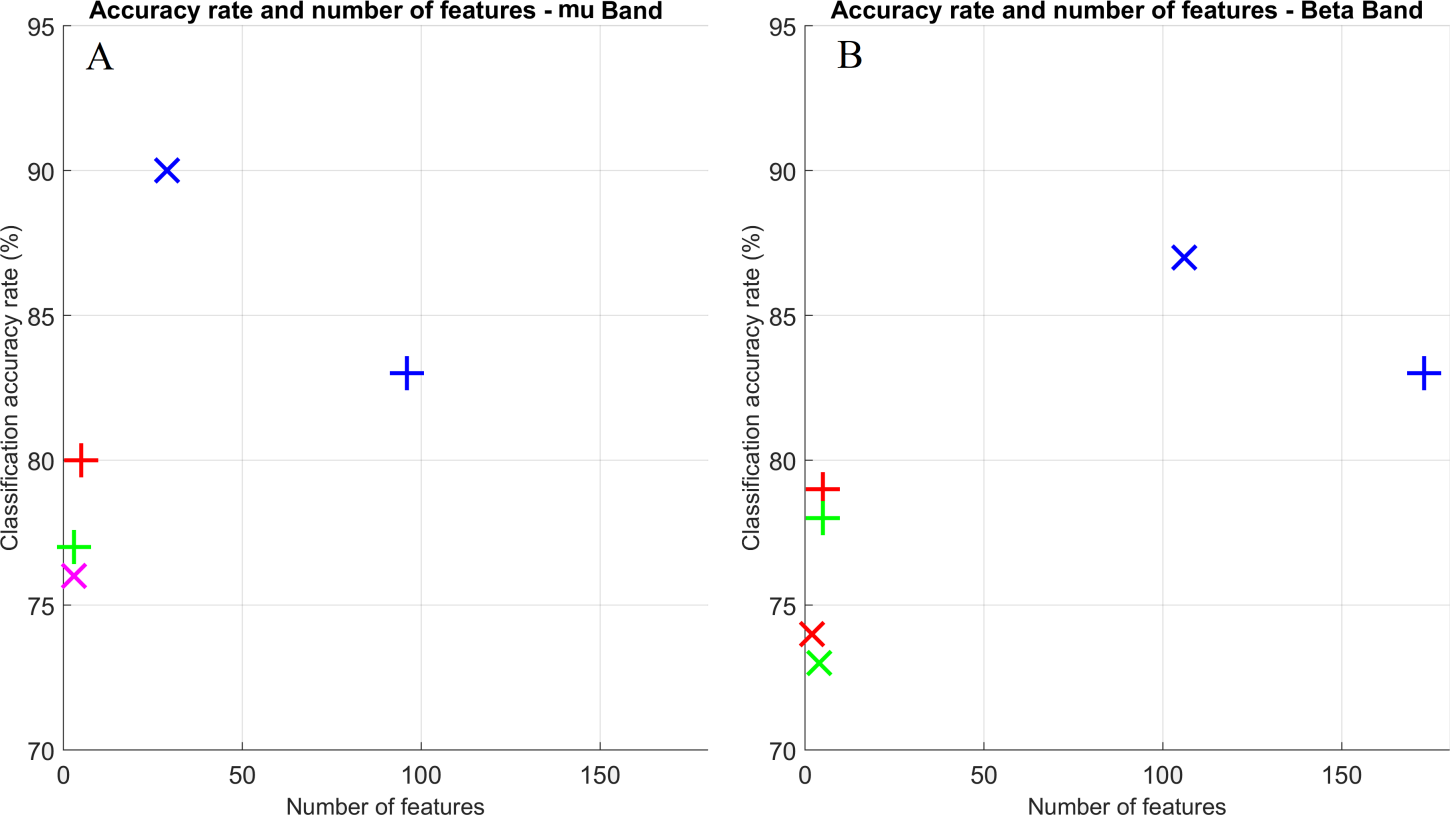
Scatter plot for classification accuracy rates vs. number of features to achieve such rates. Both frequency bands are displayed: mu (A) and beta (B). Crosses and exes indicate PSD and FCM inputs, respectively. Colors indicate the FS approach: wrapper only (blue); Pearson and wrapper combination (red); and Fisher and wrapper combination (green); overlap between Pearson and wrapper and Fisher and wrapper (magenta).

Figure 4 shows a scatter plot for the classification accuracy *vs*. number of features to achieve that rate. Crosses and exes refer to PSD and FCM inputs, respectively. The three FS strategies are displayed: use only of the wrapper (blue), Pearson filter + wrapper (red) and Fisher filter + wrapper (green). Marks in magenta are shown when an overlap between both methods occurred. From these plots, it can be seen that the best performance scenarios were obtained for the PSD method, which also provided a lesser number of features.

## Discussion

The aim of this work was two-folded: on the one hand, we wanted to find out if there was a linear relationship between ERDs and ERSs (represented by the variable Δ*PSD*) and variations in functional connectivity measures (Δ*W*). On the other hand, we wanted to verify the feasibility of using the elements of the functional connectivity matrix (*s*_*ij*_) as features for a classifier, to distinguish between left and right hands’ MI, exploring three different types of feature selection (FS) techniques.

Regarding the relationship between ERDs/ERSs and functional connectivity, we found that there is, indeed, a large number of correlations between the corresponding probed quantities (i.e., Δ*PSD* and Δ*W*), mainly for the beta band, for most electrodes, which is clearly represented by the spread of the red color for both MI tasks in Fig. 2. As a matter of a fact, for the beta band during left hand MI, some nodes achived a significant correlation for 70% of the subjects. This indicates that there is, at least to a certain extent, a reproducibility regarding which nodes best correlate to the Δ*PSD* s of MI. The number of subjects, however, is still small to doubtlessly make this claim. Therefore, further investigation on this matter would be necessary for that. The node degree is a measure of similaritiy between the recorded time series of that node and all others in the network. Hence, the fact that we were able to observe the correlations displayed in Fig. 2 suggests that MI, indeed, yielded significant alterations in the functional networks. Moreover, even though the mu band presented a much smaller number of significant correlations for all electrodes (Fig. 2), classifiction rates of both bands have very similar values (Table 1).

Although Fig. 2 locates where and how often the correlations were observed, it does not contain any information regarding how strong the relationship between the quantitities under consideration is. Thus, we computed the absolute values of the correlation for each subject (Fig. 3). We see from Fig. 3 that these values were mostly around 0.5, with the smallest being above 0.4 and the highest being above 0.9, showing a reasonable amount of correlation among ERDs and functional connectivity. In general, it can be noted that correlation strength was higher for the beta band. Also, some subjects displayed considerably larger values in this band when compared to the others (for example, subjects 5 and 7 during right hand MI; and subjects 6 and 7, during left hand MI).

From Table 1, we could observe that the *s*_*ij*_ parameters achieved a close performance to the PSD method; particularly for the beta band ((83 ± 8)% for FCM *vs.* (90 ± 8)% for PSD for the mu band, and (83 ± 7) for FCM *vs.* (87 ± 7) for PSD for the beta band). For some subjects, this even outperformed PSD features (subjects 5 and 6 for the mu band, and subjects 3 and 6 for the beta band).

Regarding the type of FS used, on average, the wrapper-only approach produced better accuracy rates for both PSD and the graph method (Table 1). In particular, for PSD features this was true with only one exception, subject 6 in the mu band, for which the wrapper + Fisher filter combination produced the best result. On the other hand, for FCM features in the mu band, there were four subjects (40%) for which the wrapper + Pearson filter combination provided the best accuracies. The influence of the chosen FS technique can be clearly observed. By simply chosing a different FS approach, average accuracy rates can vary up to 10%.

Finally, we also analyzed how many features were necessary for obtaining the average classification rates of Table 1 (Fig. 4). For both bands, accuracy rates were slightly better using FCM elements as features for the FS approaches (2) and (3) (i.e., using the wrapper combined with the Pearson and Fisher filters respectively), and with approximately the same number of features for these two methods. Nonetheless, there is a significant difference when approach (1) was performed. In addition to the accuracy rates being lower for the FCM method, it uses a considerable higher number of features; roughly twice the necessary amount for the PSD in some cases (Fig. 4A).

## Conclusion

To study the influence of the neuronal desynchronizations due to MI on the functional networks, we chose to work with the degree measure for each node, as it is a simple metric that can be easily interpreted. We hypothesized that local variations in the FCM should be accompained by modifications on the node’s degree. Thus, we quantified changes on the signal PSD due to MI when compared to rest periods (Eq. (2)) and correlated them to the corresponding variation in that node’s degree (Eq. (4)).

We found that these correlations occurred more often for the beta band, even though there is no indication of this being a decisive factor for better data classification. In fact, the obtained accuracy rates were about the same for both frequency bands.

Regarding the comparison between the connectivity method and the more traditional PSD approach, we found that, at least when analyzing purely the FCM elements, features from the PSD performed better for distinguishing hands’ MI tasks.

Although our findings indicate that MI can, in fact, alter functional networks related to this task, our strategy of using directly the *s*_*ij*_ values for data classification did not achieved the same performance as the PSD method. Therefore, we believe a further screening of relevant measures of graph topology may aid in identifying possible candidates for this task. Also, the combination of PSD and graph features should be explored in order to assess if this could bring any improvement to the classification problem.

## Additional Information and Declarations

### Data Availability

The following information was supplied regarding data availability: EEG Motor Movement/Imagery Dataset: https://www.physionet.org/pn4/eegmmidb/.
